# Off-the-Vine Ripening of Tomato Fruit Causes Alteration in the Primary Metabolite Composition

**DOI:** 10.3390/metabo3040967

**Published:** 2013-10-16

**Authors:** Augusto Sorrequieta, Luciano A. Abriata, Silvana B. Boggio, Estela M. Valle

**Affiliations:** 1 Instituto de Biología Molecular y Celular de Rosario (IBR-CONICET-UNR), Ocampo y Esmeralda, Predio CCT, Rosario 2000, Argentina; E-Mails: asorrequieta@gmail.com (A.S.); boggio@ibr-conicet.gov.ar (S.B.B.); 2 Laboratory of Biomolecular Modeling, EPFL and Swiss Institute of Bioinformatics, Lausanne 1015, Switzerland; E-Mail: luciano.abriata@epfl.ch

**Keywords:** amino acids, glutamate, ripening, tomato fruit

## Abstract

The influence of postharvest fruit ripening in the composition of metabolites, transcripts and enzymes in tomato (*Solanum lycopersicum* L.) is poorly understood. The goal of this work was to study the changes in the metabolite composition of the tomato fruit ripened off-the-vine using the cultivar Micro-Tom as model system. Proton nuclear magnetic resonance (^1^H NMR) was used for analysis of the metabolic profile of tomato fruits ripened on- and off-the-vine. Significant differences under both ripening conditions were observed principally in the contents of fructose, glucose, aspartate and glutamate. Transcript levels and enzyme activities of  -amino butyrate transaminase (EC 2.6.1.19) and glutamate decarboxylase (EC 4.1.1.15) showed differences in fruits ripened under these two conditions. These data indicate that the contents of metabolites involved in primary metabolism, and conferring the palatable properties of fruits, are altered when fruits are ripened off-the-vine.

## 1. Introduction

Ripening is a distinct process of fleshy fruit that precedes senescence [1]. Tomato (*Solanum lycopersicum* L.) fruit ripening starts when the fruit reaches the final size at the mature green stage and is completed when the fruit is red [2]. The ripening process makes tomato fruit of cultivated varieties palatable, with taste playing a major role due to changes in the content of several molecules such as sugars, organic acids and amino acids [3]. This transition is visualized when the ripening fruits turn red as lycopene and carotene accumulate [4]. Subsequently, degradation of cell walls occurs in the postharvest shelf life of red fruit, which shows high levels of free mannose [5]. Mature green fruit is also able to ripen off-the-vine, that is, when it is picked and stored on shelf, separated from the plant, and it changes the pigment contents. This is a common commercial practice in harvesting tomato fruit for human consumption, although there is a general belief that the quality of tomatoes ripened on-the-vine is better than that of fruits ripened off-the-vine. It is expected that the chemical composition of fruit ripened off-the-vine would be affected due to the import restriction from the mother plant of water and nutrients, especially sugars [6]. It has been shown that tomatoes ripened on-the-vine have significantly more lycopene and β-carotene content than those ripened off-the-vine [7]. The influence of ripening conditions on the composition of other metabolites and on the activity of enzymes related to the major compounds of tomato fruits is understudied. It requires the identification and quantitation of the different chemical constituents of tomato fruits ripened on- and off-the-vine. This is not easy, due to the large number of molecules with different physicochemical properties and stabilities and the wide number of factors that affects tomato composition.

In order to explore the impact of ripening off-the-vine on the metabolic composition of tomato fruits, we have employed ^1^H nuclear magnetic resonance (NMR) to analyze the metabolic profile of tomato fruits cultivar Micro-Tom [8] ripened on- and off-the-vine. NMR is a rapid, non-destructive, high-throughput method for the identification and quantification of plant metabolites. Moreover, it allows the study of *in vivo* samples and extracts with minimal handling. Despite it is less sensitive than mass spectrometry, sensitivity is not an issue for the study of the main cellular metabolites which are present in high concentrations, allowing straightforward quantification of several metabolites in a single spectrum by comparison to an added standard. In particular, this methodology has been successfully utilized to study the metabolic profile of tomato fruits [9] and seeds [10], to assess the effect of greenhouse-growing on tomato fruit [11], and to detect the effects introduced on fruit metabolism by genetic modifications [12,13].

In the current study we analyze by ^1^H NMR the metabolic profiles of tomato fruits ripened on- and off-the-vine. Additionally, we investigated the metabolism of glutamate, which is one of the major free amino acids of red tomato fruit [14], and a strong flavor enhancer. Glutamate is metabolized in the cytosol by the calcium/calmodulin-dependent glutamate decarboxylase (GAD; EC 4.1.1.15) rendering γ-aminobutyrate (GABA), which is catabolized to succinic semialdehyde (SSA) by the GABA transaminase (GABA-T; EC 2.6.1.19) reaction in the mitochondria [15]. SSA can be further metabolized to succinate by SSA dehydrogenase (SSADH; EC 1.2.1.16). These three enzymes constitute the GABA shunt, a metabolic pathway located at the crossing between central and secondary metabolic networks [16]. Moreover, glutamate dehydrogenase (GDH; EC 1.4.1.3), another mitochondrial enzyme, catalyzes a reversible amination/deamination reaction leading to the synthesis or the catabolism of glutamate [17]. A coordinated regulation of the gene expression of GDH and the GABA shunt might thus represent a key regulatory factor in carbon and nitrogen partitioning. Most of these enzymes have being detected in tomato fruit [14,18]. Therefore, we have also tested transcript and activities of the enzymes involved in the metabolism of glutamate and GABA in tomato fruits ripened under both ripening conditions.

## 2. Results and Discussion

### 2.1. ^1^H NMR Spectra of Mature Tomato Fruits

**Figure 1:**
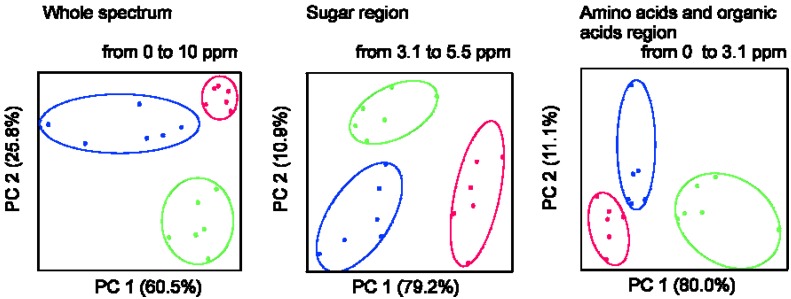
Principal component analysis (PCA) of ^1^H NMR spectra of tomato fruit. ^1^H NMR spectra of samples of pericarp extract of green (green line), red ripened on-the-vine (red line) and red ripened off-the-vine (blue line) Micro-Tom fruits were analyzed considering the whole spectra (each spectrum was normalized to a maximum intensity of 1, the water region was removed) and distinct spectral regions.

In order to analyze the complete metabolic profiles upon ripening under different conditions, ^1^H NMR spectra were collected on pericarp samples of mature green fruits and mature red fruits ripened on- and off-the-vine. Interestingly, principal component analysis (PCA) analysis performed on the whole spectra results in classification of the different samples revealing that fruits ripened off-the-vine are different from fruits ripened on-the-vine, and both different from green fruits (Figure 1). PCA analyses performed on different spectral regions show that at least those typical for sugars, amino acids and organic acids are responsible for the clustering observed (Figure 1).

A more thorough analysis of the ^1^H NMR spectra was performed by identifying and quantifying different metabolites. A program that we termed “MIXTURES” was developed *ad hoc* [19] and loaded with a dataset of NMR fingerprints for metabolites from the Biological Magnetic Resonance Database. Using this program we identified and quantified of 25 major compounds of the pericarp of three types of mature fruits (green, red ripened on-the-vine and red ripened off-the-vine) (Table 1). The metabolic profiles showed significant differences in the contents of sugars, organic acids and amino acids. Several metabolites were in significantly lower amounts in fruits ripened off-the-vine compared to fruits ripened on-the-vine (fructose, glucose, sucrose, formate, alanine, asparagine, aspartate, glutamate, phenylalanine, threonine, and tyrosine). Major quantitative differences (with more than 30% reduction) were observed in the contents of fructose, glucose, aspartate, and glutamate of fruit ripened off-the-vine. Interestingly, the content of 2-oxoglutarate was higher in this fruit than in fruit ripened on-the-vine, while the glutamate content was lower. Both compounds are connected by transaminase reactions. GABA content, instead, decreased during the transition from mature green fruit to red fruit indistinguishable from the ripening condition (Table 1).

**Table 1:** Metabolic composition of Micro-Tom mature green and red fruits ripened on- and off-the-vine. Results are presented as means of six independent experiments ± SE. Experimental data were subjected to analysis of variance (ANOVA) test (*p* < 0.05).

Metabolite	Mature green	Red ripened on-the-vine	Red ripened off-the-vine
mol/ g FW			
**Sugars**			
Fructose	64.16 ± 6.56	74.98 ± 6.71	50.76 ± 1.69*
Glucose	25.98 ± 2.63	29.51 ± 2.83	17.69 ± 1.15*
Sucrose	2.82 ± 0.63	0.15 ± 0.01	0.02 ± 0.01*
Total sugars	92.96 ± 9.28	104.63 ± 9.54	68.47 ± 2.82*
**Organic acids**			
2-oxoglutarate	5.11 ± 1.00	0.00 ± 0.00	0.99 ± 0.18*
Citrate	28.17 ± 1.02	41.96 ± 0.95	41.32 ± 0.92
Formate	0.05 ± 0.00	0.10 ± 0.01	0.08 ± 0.00*
Fumarate	0.02 ± 0.01	0.01 ± 0.00	0.01 ± 0.00
Malate	19.57 ± 2.61	4.04 ± 0.45	3.49 ± 1.05
Pyruvate	0.55 ± 0.13	0.02 ± 0.01	0.01 ± 0.00
Succinate	0.52 ± 0.13	0.00 ± 0.00	0.02 ± 0.02
Total org. acids	52.98 ± 1.04	46.13 ± 0.95	45.91 ± 1.76
**Amino acids**			
Alanine	0.61 ± 0.08	0.59 ± 0.05	0.42 ± 0.01*
Asparagine	1.06 ± 0.04	0.88 ± 0.07	0.32 ± 0.08*
Aspartate	0.51 ± 0.06	2.30 ± 0.04	1.36 ± 0.26*
GABA	5.27 ± 0.32	2.52 ± 0.39	2.73 ± 0.92
Glutamine	4.68 ± 0.17	2.49 ± 0.29	2.10 ± 0.18
Glutamate	0.78 ± 0.09	5.19 ± 0.74	3.04 ± 0.14*
Isoleucine	0.22 ± 0.04	0.09 ± 0.02	0.08 ± 0.01
Phenylalanine	0.20 ± 0.01	0.12 ± 0.02	0.05 ± 0.02*
Threonine	0.23 ± 0.05	0.30 ± 0.01	0.33 ± 0.01
Tryptophan	0.10 ± 0.02	0.11 ± 0.01	0.06 ± 0.01*
Tyrosine	0.19 ± 0.15	0.14 ± 0.01	0.07 ± 0.01*
Valine	0.27 ± 0.04	0.07 ± 0.02	0.08 ± 0.01
Total am. acids	14.13 ± 0.93	15.32 ± 1.15	10.20 ± 0.89
**Others**			
Ethanol	3.34 ± 0.53	2.30 ± 0.15	1.53 ± 0.06*
Methanol	6.56 ± 0.60	5.94 ± 0.35	4.74 ± 0.07
Trigonelline	0.17 ± 0.01	0.15 ± 0.01	0.11 ± 0.01

^*^ The asterisks indicate statistical significance of means between both ripening conditions.

**Figure 2:**
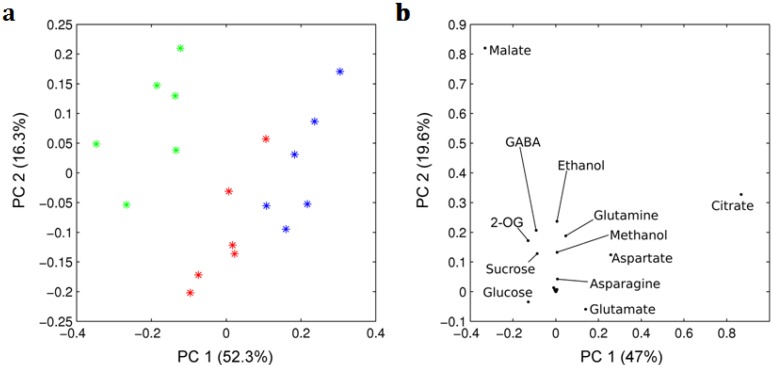
PCA analysis performed on metabolite concentrations for all the studied samples. Panels show (**a**) the scores plots, in which each point corresponds to one sample, and (**b**) the loading plots, in which each point is a metabolite concentration. Green, red and blue colors indicate maturation stage of fruits as described in Figure 1, and 2-OG indicates 2-oxoglutarate.

A PCA analysis that underscores the ripening stages was also performed on the 25 metabolites quantified in 18 samples from mature green, red ripened on-the-vine and red ripened off-the-vine fruits of different plants. The PCA scores (Figure 2a) revealed again differential clustering between the three types of fruits with the two first PCs explaining 69% of the total variability. Additionally, the loadings plot pointed out that glucose, glutamate, aspartate, malate and citrate are the metabolites that mostly differentiate among the ripening stages (Figure 2b).

Recently, biochemical characterizations of postharvest fruits were performed in another variety of tomato (*Solanum lycopersicum* L. cv Plaisance) [5]. Similar changes were observed in major compounds such as decrease in malic acid and increase in citric acid, aspartate and glutamate although they had used red fruits stored at 18 °C and gas chromatography–mass spectrometry (GC-MS) for analysis. Comparison of ^1^H NMR and GC-MS metabolic profiles of tomato fruits showed that a substantial number of significantly correlating metabolites were qualitative- and quantitatively similar in the fruits, suggesting that both platforms complement each other [20].

### 2.2. Enzymes of the GABA Shunt in Ripening Fruit

It is known that glutamate is the most abundant free amino acid of mature Micro-Tom fruit [14] and that the GABA content of green fruit decreases during ripening concomitantly with the increase in glutamate content [14,21]. To analyze the metabolism of GABA and glutamate during both ripening conditions, we determined activities of the enzymes involved in the GABA shunt, which was described to be present in tomato fruits [14,18]. From all the enzymes measured, GAD showed marked decrease in red fruit ripened on-the-vine but not in fruits ripened off-the-vine (Table 2). GABA-T instead, showed a significant increase in red fruits ripened on-the-vine but not off-the-vine. Deaminating GDH decreased sharply under both ripening conditions, and the GDH aminating to deaminating activity ratio increased as well. The SSADH activity decreased during ripening although the level was similar under both conditions. These data showed that the GABA shunt enzymes involved in glutamate synthesis (GDH and GABA-T) are less active when fruits are ripened off-the-vine than when they ripened on-the-vine, suggesting that they could be the reason for the decrease in glutamate and the increase in 2-oxoglutarate contents of red fruit ripened off-the-vine. Although the GABA content could not be correlated with higher GAD activity in red fruit ripened off-the-vine, the decrease in SSADH activity during fruit ripening is coincident with higher succinate content of mature green fruits.

**Table 2:** Enzyme activities in mature green and red fruits ripened on- and off-the-vine. One U is the amount of enzyme which catalyzes the transformation of 1 μmol of substrate per min. Results are presented as means of three independent experiments ± SE.

Enzyme	Green	Red on-the-vine mU/mg protein	Red off-the-vine
Aminating GDH	26.33 ± 5.56	25.04 ± 3.61	30.50 ± 1.71
Deaminating GDH	89.20 ± 11.71	7.22 ± 1.25	12.63 ± 2.55
GDH aminating/deaminating	0.29 ± 0.02	3.49 ± 0.10	2.49 ± 0.37
GAD	86.74 ± 15.27	5.11 ± 2.60	32.28 ± 7.72*
GABA-T	8.30 ± 3.00	47.15 ± 1.25	13.19 ± 0.28*
SSADH	9.88 ± 0.65	1.50 ± 0.47	2.25 ± 0.74

^*^ The asterisks indicate statistical significance of means between both ripening conditions.

From all the transcripts of the GABA shunt genes only *GAD1* and *GABA-T3* varied significantly under both ripening conditions (Table 3). However, the lower level of *GAD1* transcripts in fruit ripened off-the-vine did not follow the GAD activity changes observed under both ripening conditions (Table 2). These results indicate that higher GAD activity in fruit ripened off-the-vine than ripened on-the-vine is not due to the transcript levels, suggesting a translational or post-translational regulation of *GAD* expression in tomato fruit. Previously, it was reported [18] that Micro-Tom fruits dramatically decreased GAD activity during ripening correlating with levels of *GAD2* and *GAD3* but not with *GAD1*. Moreover, *GAD2* and *GAD3* expression were enhanced by salinity in Micro-Tom fruits, but salt stress did not affect GAD enzymatic activity [21]. Poor correlation between transcript levels and enzyme activity were already reported in tomato during fruit development [22], suggesting that this is a consequence of the complex networks that link events at different functional levels. On the other hand, the repression of *GABA-T3* and the lower GABA-T activity in the red off-the-vine fruit are probably involved in the lower accumulation of glutamate in this fruit.

**Figure 3:**
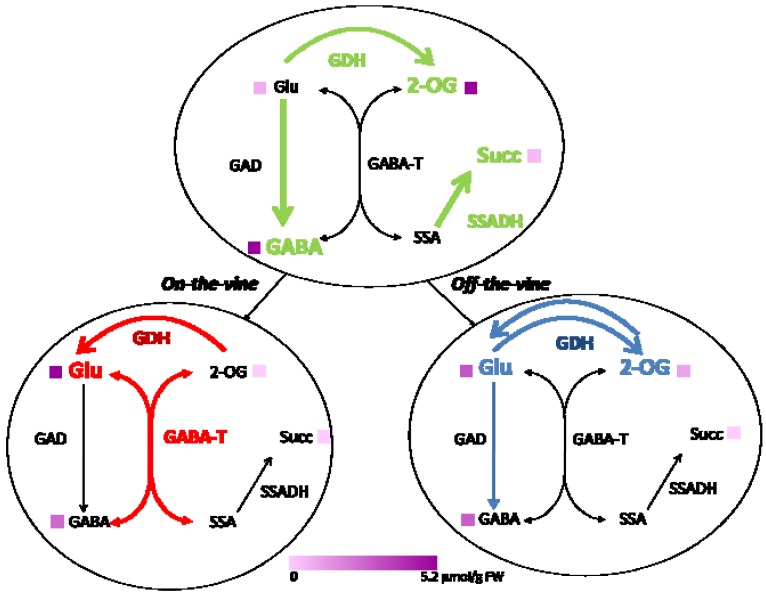
Metabolism of glutamate and GABA in tomato fruit. Enzymes and metabolites measured in mature green fruit (upper part), and fruits ripened on- and off-the-vine (lower part) are shown. Colored arrows and letters indicate increased levels in mature green fruit (green), on-the-vine (red) or off-the-vine (blue). Thickness of arrows and size of letters are proportional to corresponding reaction activity (taken from Table 2) and metabolite accumulation, respectively. At the bottom, a violet scale representing the content of each metabolite (taken from Table 1) is shown.

A simplified scheme showing the variation in the metabolism of glutamate and GABA of mature green and red fruits ripened on- and off-the-vine is presented in Figure 3. GABA, 2-oxoglutarate and succinate were significantly higher in mature green fruit than in red fruits, independently of the ripening condition. These metabolites are the product of the reactions catalyzed by GAD and SSADH detected in mature green fruit. Glutamate, which is the predominant free amino acid of red fruit, was significantly lower in fruits ripened off-the-vine than on-the-vine. This decrease in glutamate level in red fruit ripened off-the-vine (Table 1) could be due to the statistically significant changes in the activities of GAD (higher) and GABA-T (lower) in this fruit compared to red fruit ripened on-the-vine (Table 2).

**Table 3:** Expression of genes encoding γ-aminobutyrate (GABA) shunt enzymes in mature fruits harvested after on- and off-the-vine ripening. Fruits were collected at mature green stage and red ripened on- and off-the-vine for transcript profile analysis by qRT-PCR. Transcript levels are relative to those in mature green fruit. Results are the means of three independent fruit samples ± SE. The experimental data were subjected to ANOVA followed by Holm Sidak test analysis (*p* < 0.05).

Gene	Green	Red on-the-vine	Red off-the-vine
*GAD1*	1.025 ± 0.227	0.806 ± 0.253	0.087 ± 0.011*
*GAD2*	1.012 ± 0.153	0.130 ± 0.047	0.015 ± 0.008
*GAD3*	1.036 ± 0.270	0.240 ± 0.006	0.198 ± 0.079
*GABA-T1*	1.124 ± 0.512	0.089 ± 0.016	0.083 ± 0.010
*GABA-T2*	1.000 ± 0.021	0.010 ± 0.004	0.004 ± 0.002
*GABA-T3*	1.021 ± 0.206	0.680 ± 0.210	0.318 ± 0.004*
*SSADH*	1.001 ± 0.069	2.028 ± 0.334	2.815 ± 0.596
*GDHA1*	1.026 ± 0.209	0.625 ± 0.077	0.503 ± 0.103
*GADHB*	1.007 ± 0.115	0.359 ± 0.085	0.388 ± 0.020

^*^ Asterisks indicate significant data between both ripening conditions.

## 3. Experimental Section

### 3.1. Plant Material and Ripening Conditions

Tomato (cv. Micro-Tom) seeds were provided by Gulf Coast Research and Education Center, University of Florida, USA. The seeds were germinated in soil, and Micro-Tom plants were grown in a controlled environment cabinet under a light intensity at the top of a fruit-containing plant of 400 µmol s^−1^m^−2^. The temperature ranged from 23 °C during the light period (14 h) to 18 °C in the dark, and the relative humidity was 70%. Plants were grown in soil, continuously maintained under optimal irrigation and supplied weekly with half strength Hoagland solution [23]. When fruits reached mature green stage, they were ripened naturally on the plant (on-the-vine fruit) or picked and directly placed on a shelf and stored at the growing conditions (off-the-vine fruit). Fruits were harvested at the mature green stage, or red stages. Pericarp tissue of the harvested fruits was obtained by removing the locule tissues and seeds and immediately processed or frozen in liquid nitrogen and stored at –80 °C until analysis.

### 3.2. NMR Spectroscopy

Liquid nitrogen powder (1 g) from each sample was rapidly dissolved in cold 0.3 mL of 1 M sodium phosphate buffer (pH 7.4) prepared in D_2_O to obtain a mixture containing about 30% by weight of D_2_O. The mixtures were centrifuged at 13,500 rpm for 15 min at 4 °C and the supernatant filtered to remove any insoluble material. One mM of internal standard (TSP: 3-(trimethylsilyl) propionic-2,2,3,3-d_4_ acid) was added to the resulting transparent soluble fraction and the solution was subjected to spectral analysis at 600.13 MHz on a Bruker Avance II spectrometer. Proton spectra were acquired at 298 K by adding 512 transients of 32K data points with a relaxation delay of 5 s. A 1D-NOESY pulse sequence was utilized to remove the water signal. The 90° flip angle pulse was always ~10 μs. Proton spectra were referenced to the TSP signal (δ = 0 ppm) and their intensities were scaled to that of TSP. Spectral assignment and identification of specific metabolites was established by fitting the reference ^1^H NMR spectra of several compounds using the software Mixtures, developed *ad hoc* as an alternative to commercial programs [19]. Further confirmation of the assignments for some metabolites was obtained by acquisition of new spectra after addition of authentic standards.

### 3.3. Enzyme Extraction and Analysis

For the extraction of GDH, GAD, GABA-T and SSADH activities 1 g of frozen tissue was ground in 0.3 mL of 0.75 M Tricine–KOH (pH 8), 100 mM MgCl_2_, 10 mM EDTA, 50 mM β-mercaptoethanol, 15 mM phenylmethylsulfonylfluoride, and 0.3 mL of glycerol and 2% (w/v) polyvinylpolypyrrolidone, and for GABA-T 1 mM pyridoxal phosphate was also added. The supernatants were desalted using Sephadex G-25 columns equilibrated with the same extraction buffer (10 times diluted) plus 10% (v/v) glycerol. GDH, GAD and GABA-T activities were determined as previously described [14]. The SSADH activity was measured at 30 °C as previously described [24] with slight modifications. The assay mixture contained 0.2 M glycine (pH 9.5); 14 mM β-mercaptoethanol; 0.5 mM NAD^+^ and 0.1 mM SSA. The reaction was started by addition of SSA and followed by the increase in absorbance at 340 nm (molar extinction coefficient of NADH: 6,220 M^−1^ cm^−1^). One U is the amount of enzyme which catalyzes the transformation of 1 μmol of substrate per min. Protein concentrations of enzyme extracts were determined according to [25] using bovine serum albumin as standard.

### 3.4. Total RNA Extraction and Real-Time

*PCR analysis.* Total RNA was extracted from frozen pericarps, treated with DNAse and used as template for cDNA synthesis with an oligo(dT) primer according to [14]. Real-time PCR (qPCR) reactions were performed in a Mastercycler^®^ ep realplex thermal cycler (Eppendorf, Westbury, USA) using the intercalation dye SYBR Green I (Roche). Transcript levels of *GDH*, *GAD*, *GABA-T* and *SSADH* genes in the RNA samples were normalized with transcript levels of *RPL2* (encoding the ribosomal protein large subunit 2). PCR mixture composition, PCR program and data analysis was carried out as previously described [14]. Gene-specific primers used were as follows: *SlGDH-NAD; A1-3* [26], forward 5'-CCAGACATCTATGCCAATGC-3’ and reverse 5'-ATTCACCCCCAATGTGAATG-3'; *SlGDH-NAD;B1* (GenBank accession AF403178), forward 5'-AAGGAGTCACCATCCTACCG-3' and reverse 5'-TGTGAGTCTTGCACATATCCTTG-3'; *GAD1* (GenBank accession AB359913), forward 5'-GATTTCAGCCACAGCCTAGC-3' and reverse 5'-CTGCGATTTTCCTCCAATGT -3'; *GAD2* (GenBank accession AB359914), forward 5'-AGCGACTGGTTATGGACATC-3' and reverse 5'-GCAACAAACTTCTTCCATGC-3'; *GAD3* (GenBank accession AB359915), forward 5'-AGAGAGGACTTCTCCCGAAC-3' and reverse 5'-ACATATTTCTTCCAAAACTCAGC-3'; *GABA-T1* (GenBank accession AY240229), forward 5'-CCTTGCCACAGAGTTTGCG-3' and reverse 5'-CAAGTTCTTCTGGAGTAACTAC-3'; *GABA-T2* (GenBank accession AY240230), forward 5'-GTACTTTCTACAGAGTTTGTAG-3' and reverse 5'-AGTTCTTCAAGACTCAAGGTG-3'; *GABA-T3* (GenBank accession AY240231), forward 5'-TGAGAAGCATGGAGTGTTGG-3' and reverse 5'-TCATGAGCTTTTTATCTTCTTCTGA-3'; *SSADH* (GenBank accession AB359921), forward 5'-AGCAATCCAAATGGCTAACG-3' and reverse 5'-AATGGAGCTACCTCGGTTGA-3', and *RpL2* (GenBank accession X64562), forward 5'-CGTGGTGTTGCTATGAATCC-3' and reverse 5'-GTCAGCTTTGGCAGCAGTAG-3'. qPCR for each gene was done on 3 biological replicates. To ensure amplification of one specific gene product melting curves were performed as previously described [14]. Additionally, the identity of the amplified fragments was confirmed by nucleotide sequencing. PCR conditions were 1 min at 95 °C and 40 cycles of 15 s at 95 °C, 30 s at 55 °C and 40 s at 72 °C. Following amplification, products were denatured by heating from 60 to 95 °C to check amplification specificity. Real time was performed using a SYBR Green fluorescence-based assay. Gene specific cDNA amounts were calculated from threshold cycle (*Ct)* values, expressed as relative to controls, and normalized with respect to *RPL2* cDNA, used as internal reference. Values were normalized by an internal reference (*Ct_r_*) according to the equation ∆*Ct* = *Ct* − *Ct_r_* and quantified as 2^−∆*Ct*^. A second normalization by a control (*Ct_c_*) ∆∆*Ct* = *Ct* – *Ct_c_* produces a relative quantification: 2^−∆∆*Ct*^ [27].

### 3.5. Statistical Methods

The experimental data were subjected to analysis of variance (ANOVA). When F-test data were significant in ANOVA, individual means were further tested by LSD. A p value less than 0.05 was considered statistically significant. Models assumptions were tested by analysis of residuals. Processed and scaled ^1^H NMR spectra were binned into 0.01 ppm segments and this data was subjected to statistical analysis by using MATLAB version 7.4 [28] (MathWorks, Inc., Natick MA) scripts. The mean comparison between lines for one stage was determined by Student’s *t*-test using SAS software version 8.01 [28].

## 4. Conclusions

Our ^1^H NMR results showed significant differences in the metabolic profiles of fruits ripened on- and off-the-vine. The contents of metabolites involved in primary metabolism, and conferring the palatable properties of fruits, are altered when fruits are ripened off-the-vine. The consequence of this is the inferior quality of tomato fruits ripened off-the-vine due in part to the lower levels of fructose and glucose sucrose, which are involved in conferring the sweet taste to fruit, together with aspartate and glutamate, both implicated in UMAMI taste, and other compounds present at low concentrations.

## References

[1] Gapper N.E., McQuinn R.P., Giovannoni J.J. (2013). Molecular and genetic regulation of fruit ripening. Plant Mol. Biol..

[2] Gillaspy G., Ben-David H., Gruissem W. (1993). Fruits: A Developmental Perspective. Plant Cell..

[3] Carrari F., Fernie A.R. (2006). Metabolic regulation underlying tomato fruit development. J. Exp. Bot..

[4] Bortolotti S., Boggio S.B., Delgado L., Orellano E.G., Valle E.M. (2003). Different induction patterns of glutamate metabolising enzymes in ripening fruits of the tomato mutant *green flesh*. Physiol. Plant..

[5] Oms-Oliu G., Hertog M.L.A.T.M., van de Poel B., Ampofo-Asiama J., Geeraerd A.H., Nicolaï B.M. (2011). Metabolic characterization of tomato fruit during preharvest development, ripening, and postharvest shelf-life. Postharvest Biol. Technol..

[6] Beckles D.M. (2012). Factors affecting the postharvest soluble solids and sugar content of tomato (Solanum lycopersicum L.) fruit. Postharvest Biol. Technol..

[7] Malacrida C., Valle E.M., Boggio S.B. (2006). Postharvest chilling induces oxidative stress response in the dwarf tomato cultivar Micro-Tom. Physiol. Plant..

[8] Scott J.M., Harbaugh B.K. (1989). Micro-Tom: a miniature dwarf tomato. Florida Agr. Expt. Sta. Circ..

[9] Sobolev A.P., Segre A., Lamanna R. (2003). Proton high-field NMR study of tomato juice. Magn. Reson. Chem..

[10] Mounet F., Lemaire-Chamley M., Maucourt M., Cabasson C., Giraudel J.L., Deborde C., Lessire R., Gallusci P., Bertrand A., Gaudillere M. (2007). Quantitative metabolic profiles of tomato flesh and seeds during fruit development: complementary analysis with ANN and PCA. Metabolomics.

[11] Deborde C., Maucourt M., Baldet P., Bernillon S., Biais B., Talon G., Ferrand C., Jacob D., Ferry-Dumazet H., de Daruvar A. (2009). Proton NMR quantitative profiling for quality assessment of greenhouse-grown tomato fruit. Metabolomics.

[12] Le Gall G., Colquhoun I.J., Davis A.L., Collins G.J., Verhoeyen M.E. (2003). Metabolite profiling of tomato (*Lycopersicon esculentum*) using ^1^H NMR spectroscopy as a tool to detect potential unintended effects following a genetic modification. J. Agric. Food Chem..

[13] Mattoo A.K., Sobolev A.P., Neelam A., Goyal R.K., Handa A.K., Segre A.L. (2006). Nuclear magnetic resonance spectroscopy-based metabolite profiling of transgenic tomato fruit engineered to accumulate spermidine and spermine reveals enhanced anabolic and nitrogen-carbon interactions. Plant Physiol..

[14] Sorrequieta A., Ferraro G., Boggio S.B., Valle E.M. (2010). Free amino acid production during tomato fruit ripening: a focus on L-glutamate. Amino Acids.

[15] Clark S.M., Di Leo R., van Cauwenberghe O.R., Mullen R.T., Shelp B.J. (2009). Subcellular localization and expression of multiple tomato gamma-aminobutyrate transaminases that utilize both pyruvate and glyoxylate. J. Exp. Bot..

[16] Fait A., Fromm H., Walter D., Galili G., Fernie A.R. (2008). Highway or byway: the metabolic role of the GABA shunt in plants. Trends Plant Sci..

[17] Forde B.G., Lea P.J. (2007). Glutamate in plants: metabolism, regulation, and signalling. J. Exp. Bot..

[18] Akihiro T., Koike S., Tani R., Tominaga T., Watanabe S., Iijima Y., Aoki K., Shibata D., Ashihara H., Matsukura C. (2008). Biochemical mechanism on GABA accumulation during fruit development in tomato. Plant Cell Physiol..

[19] Abriata L.A. (2012). Utilization of NMR spectroscopy to study biological fluids and metabolic processes: Two introductory activities. Conc. Magn. Reson. Part. A.

[20] Zanor M.I., Lopez M., Boggio S., Sorrequieta A., Pratta G., Rodriguez G.R., Zorzoli R., Picardi L.A., Carrari F., Valle E.M. (2010). Comparison of ^1^H-NMR and GC-MS metabolic profile of tomato fruits from a breeding program progeny. Biocell.

[21] Yin Y.G., Tominaga T., Iijima Y., Aoki K., Shibata D., Ashihara H., Nishimura S., Ezura H., Matsukura C. (2010). Metabolic alterations in organic acids and gamma-aminobutyric acid in developing tomato (*Solanum lycopersicum* L.) fruits. Plant Cell Physiol..

[22] Steinhauser M.C., Steinhauser D., Koehl K., Carrari F., Gibon Y., Fernie A.R., Stitt M. (2010). Enzyme activity profiles during fruit development in tomato cultivars and *Solanum. pennellii*. Plant Physiol..

[23] Scarpeci T.E., Marro M.L., Bortolotti S., Boggio S.B., Valle E.M. (2007). Plant nutritional status modulates glutamine synthetase levels in ripe tomatoes (*Solanum lycopersicum* cv. Micro-Tom). Plant Physiol..

[24] Breitkreuz K.E., Shelp B.J. (1995). Subcellular compartmentation of the 4-aminobutyrate shunt in protoplasts from developing soybean cotyledons. Plant Physiol..

[25] Bradford M.M. (1976). A rapid and sensitive method for the quantitation of microgram quantities of protein utilizing the principle of protein-dye binding. Anal. Biochem..

[26] Ferraro G., Bortolotti S., Mortera P., Schlereth A., Stitt M., Carrari F., Kamenetzky L., Valle E.M. (2012). Novel glutamate dehydrogenase genes show increased transcript and protein abundances in mature tomato fruits. Plant Physiol..

[27] Livak K.J., Schmittgen T.D. (2001). Analysis of relative gene expression data using real-time quantitative PCR and the 2(-Delta Delta C(T)) Method. Methods.

[28] Neily M.H., Matsukuraa C., Maucourt M., Bernillon S., Deborde C., Moing A., Yin Y., Saito T., Mori K., Asamizu E. (2011). Enhanced polyamine accumulation alters carotenoid metabolism at the transcriptional level in tomato fruit over-expressing spermidine synthase. J. Plant Physiol..

